# Health insurance and kidney transplantation outcomes in the United States: a systematic review and AI-driven analysis of disparities in access and survival

**DOI:** 10.1080/0886022X.2025.2513007

**Published:** 2025-06-09

**Authors:** Oscar A. Garcia Valencia, Supawadee Suppadungsuk, Charat Thongprayoon, Yuh-Shan Ho, Noppachai Siranart, Wannasit Wathanavasin, Caroline C. Jadlowiec, Shennen A. Mao, Napat Leeaphorn, Karim M. Soliman, Hatem Ali, Pooja Budhiraja, Jing Miao, Wisit Cheungpasitporn

**Affiliations:** a Division of Nephrology and Hypertension, Department of Medicine, Mayo Clinic, Rochester, MN, USA; b Chakri Naruebodindra Medical Institute, Faculty of Medicine, Ramathibodi Hospital, Mahidol University, Bangkok, Thailand; c Trend Research Centre, Asia University, Wufeng, Taiwan; d Nephrology Unit, Department of Medicine, Charoenkrung Pracharak Hospital, Bangkok Metropolitan Administration, Bangkok, Thailand; e Division of Transplant Surgery, Department of Surgery, Mayo Clinic, Pheonix, AZ, USA; f Division of Transplant Surgery, Department of Surgery, Mayo Clinic, Jacksonville, FL, USA; g Department of Medicine, Division of Nephrology, Medical University of South Carolina, Charleston, SC, USA; h Medical Services, Ralph H. Johnson VA Medical Center, Charleston, SC, USA; i Renal and Transplant Department, University Hospitals of Wales, Cardiff, UK; j Division of Nephrology and Hypertension, Department of Medicine, Mayo Clinic, Pheonix, AZ, USA

**Keywords:** Kidney transplantation, health equity, insurance disparities, Medicare and Medicaid, transplant outcomes, artificial intelligence

## Abstract

**Background:**

Kidney transplantation is the preferred treatment for end-stage kidney disease (ESKD) in the United States, yet access and outcomes vary by insurance type, race, and socioeconomic status. This systematic review synthesizes U.S.-based evidence on how insurance coverage influences transplant waitlisting, access, and outcomes. AI-assisted analysis was used to quantify disparities and propose policy recommendations.

**Methods:**

A systematic review of MEDLINE, EMBASE, and the Cochrane Database (through November 2024) was conducted to identify studies on insurance-related disparities in U.S. kidney transplantation (PROSPERO: CRD42023484733). AI-assisted synthesis using o3-mini-high (2025) was employed to identify patterns and guide policy development.

**Results:**

Among 2,163 records, 14 studies met inclusion criteria. Patients with Medicare or Medicaid—particularly racial and ethnic minorities—had lower referral rates and higher transplant waitlist rejection compared to those with private insurance. Socioeconomic barriers such as low income and limited education further impaired access and worsened post-transplant outcomes. Publicly insured recipients had higher post-transplant mortality and graft failure rates. Loss of Medicare after 36 months was associated with reduced immunosuppressant adherence and increased rejection. Disparities were amplified by Medicaid expansion variability and inconsistent transplant center policies. AI-assisted analysis confirmed these disparities and generated policy proposals including standardized referral guidelines, lifelong immunosuppressant coverage, targeted financial aid, equity-linked incentives for transplant centers, and scalable digital health solutions.

**Conclusion:**

Insurance type, race, and socioeconomic status significantly influence kidney transplant access and outcomes. AI-assisted analysis identified structural inequities and informed targeted policy strategies to advance transplant equity and support broader healthcare reform.

## Introduction

Kidney transplantation is the preferred treatment for end-stage kidney disease (ESKD), offering superior quality of life and survival rates compared to long-term dialysis [1–3]. However, access to kidney transplantation in the United States remain inequitable, with significant disparities driven by insurance type, race, and socioeconomic status [4,5]. These factors influence every key aspect of the transplant care cascade—from referral and evaluation to waitlisting and post-transplant management and outcomes [6,7].

Patients covered by public insurance programs, such as Medicaid, often experience delayed referrals, prolonged durations of pre-listing dialysis, and lower rates of successful transplantation compared to those with private insurance [8,9]. Recent data from the 2023 OPTN/SRTR Annual Data Report highlight significant insurance-based disparities in kidney transplantation. Among deceased donor kidney transplant (DDKT) recipients, 61.7% were Medicare beneficiaries compared to only 37.8% of living donor kidney transplant (LDKT) recipients. Furthermore, 54.1% of LDKT recipients had private insurance, while only 27.3% of DDKT recipients did, underscoring persistent inequities in access to living donor transplants [10].

These disparities disproportionately affect racial and ethnic minorities, particularly Black, Hispanic, and American Indian/Alaska Native patients, who are more likely to be publicly insured and face compounded barriers from adverse social determinants of health [11–13]. The current hybrid U.S. healthcare framework—comprising both public and private insurance systems—further complicates efforts to ensure equitable transplant access and outcomes [14]. Despite substantial literature documenting disparities in kidney transplantation, major gaps remain in systematically translating these findings into structured, reproducible, and scalable policy solutions [15–17]. Traditional approaches, often reliant on narrative synthesis and expert opinion, may introduce variability, subjective prioritization, and inconsistent policy framing across studies and institutions [15–18]. There is a critical need for methodologies that can operationalize complex evidence patterns into standardized, transparent, and data-driven policy frameworks to enhance health equity in transplantation.

Emergent advancements in artificial intelligence (AI) offer novel opportunities to address persistent inequities in kidney transplantation by enhancing the identification, quantification, and policy response to social determinants of health [18,19]. While prior studies have highlighted disparities linked to race, income, and insurance status [15–17], our work systematically synthesizes this evidence and uniquely integrates AI-driven policy modeling to propose actionable solutions [18]. AI-assisted analyses have enabled a more granular understanding of how insurance type intersects with race and socioeconomic factors to affect transplant outcomes. These methodologies not only enhance our understanding of disparities but also inform targeted policy solutions ultimately dismantling systemic barriers. Proposed interventions specifically address insurance-related disparities, including the expansion of public and private insurance coverage to support pre-transplant evaluations, lifelong immunosuppressive therapy, and ancillary services such as community health workers and caregiver support programs. By proposing that public insurance programs (e.g., Medicaid) fund such services, these interventions directly align with insurance policy reform, aiming to mitigate the systemic barriers faced by publicly insured patients [18,20,21].

Given the pressing need for a comprehensive evaluation, this study aims to systematically review and compare the impact of different insurance types on kidney transplantation accessibility and outcomes. By integrating AI-driven insights with traditional epidemiological analyses, we seek to develop a scalable framework for healthcare policy reform—one that not only addresses disparities in kidney transplantation, but also informs broader health equity initiatives.

## Methods

### Systematic review registration and search strategy

This systematic review was conducted in accordance with PRISMA guidelines and was registered with PROSPERO (CRD42023484733). A comprehensive literature search was conducted using MEDLINE, EMBASE, and The Cochrane Database of Systematic Reviews through November 2024. The search terms included ‘renal transplantation’, ‘kidney transplantation’, ‘end-stage renal disease (ESRD)’, ‘end-stage kidney disease (ESKD)’, ‘renal replacement therapy (RRT)’, ‘kidney replacement therapy (KRT)’, ‘Medicare’, ‘Medicaid’, and ‘private insurance’. Historical terminology such as ‘renal’, ‘ESRD’, and ‘RRT’ was retained in the search strategy to ensure complete retrieval of relevant studies published under earlier nomenclature [22]. Updated KDIGO-recommended terminology (‘kidney’, ‘ESKD’, ‘KRT’) is used throughout the manuscript outside of the search strategy for consistency with contemporary standards. The search strategy was developed in collaboration with an experienced Mayo Clinic librarian to ensure methodological rigor.

### Eligibility criteria and study selection

This review followed the PICO framework**:**

- Population: Individuals in the United States undergoing kidney transplantation.
- Intervention: Exposure to different insurance types (Medicare, Medicaid, and private insurance).
- Comparison: Comparative outcomes among different insurance coverage groups.
- Outcomes: Primary outcomes included waitlisting rates, access to transplantation, post-transplant survival, allograft failure, and medication adherence.

Eligible studies included observational studies, cohort studies, and clinical trials that examined the impact of insurance type on kidney transplantation outcomes in the United States. The search encompassed studies from the earliest available records in each database through November 2024, with no language restrictions.

### Study selection process

Two independent reviewers (O.A.G.V. and S.S.) screened titles and abstracts, followed by full-text assessments. Discrepancies were resolved through consensus discussions.

### Information sources and supplementary searches

- **Primary Databases:** MEDLINE, EMBASE, and The Cochrane Database of Systematic Reviews.
- **Search Period:** From database inception to November 2024.
- **Search Strategy:** A structured Boolean logic approach combining MeSH terms and **keywords** related to kidney transplantation and insurance coverage (e.g., Medicare, Medicaid, private insurance). No language restrictions were applied.
- **Manual Reference Screening:** Additional relevant articles were identified through **hand-searching** reference lists of key studies.
- **Supplementary Materials:** Full search strategies are provided in the **Supplementary Data** section.

### Data collection and extraction

Two independent reviewers (O.A.G.V. and S.S.) screened titles and abstracts, followed by full-text assessments for eligibility. Inter-reviewer agreement during both the title/abstract screening and the full-text review phases was quantified using Cohen’s kappa statistic. The kappa value for title and abstract screening was 0.81 (indicating excellent agreement), and for full-text review was 0.85 (indicating excellent agreement).

Similarly, during the data extraction phase, two independent reviewers extracted data using a standardized data collection form. The inter-reviewer agreement for key extracted variables (study design, population characteristics, insurance type, primary and secondary outcomes) was assessed with a kappa statistic of 0.83, demonstrating excellent reliability.

Any discrepancies between reviewers at either the study selection or data extraction stages were resolved through consensus discussions. If consensus could not be reached, a third senior investigator (W.C.) was consulted to adjudicate disagreements. However, no adjudication was ultimately required, as consensus was achieved in all cases.

Outcomes of Interest

The primary outcomes of this systematic review were:

- Access to kidney transplantation, measured by waitlisting rates and transplantation rates across different insurance types (Medicare, Medicaid, private insurance).
- Post-transplant clinical outcomes, specifically patient survival and allograft survival (graft failure rates).

The secondary outcomes included:

- Medication adherence post-transplant, particularly immunosuppressive medication adherence, and its association with insurance status.
- Time to transplantation, including duration of dialysis prior to listing or transplant.
- Rates and causes of waitlist removal (e.g., death, medical unsuitability) by insurance category.
- Socioeconomic and demographic factors modifying the association between insurance type and transplant outcomes.

The Newcastle-Ottawa Scale (NOS) [23] was used to assess the methodological quality of observational studies by evaluating three key areas: selection bias, which refers to the representativeness of the study populations; comparability, which assesses the adjustment for potential confounders; and outcome assessment, which evaluates the validity of outcome measurement and the adequacy of follow-up.

For cohort studies, a NOS score of less than five was classified as low quality, a score between five and six was considered fair quality, and a score greater than six was categorized as high quality [24]. For cross-sectional studies, a NOS score of less than four was deemed unsatisfactory, a score between five and six was considered satisfactory, a score between seven and eight was classified as good, and a score between nine and ten was categorized as very good [24].

Two independent reviewers (O.A.G.V. and S.S.) conducted the quality assessments, resolving any discrepancies through consensus discussions.

### Integration of AI-assisted analysis

In this study, AI was not utilized to perform literature search, selection, or data extraction, which remained fully manual and consistent with PRISMA standards. Instead, the AI model (o3-mini-high, 2025) was applied after manual evidence synthesis to assist in policy generation [25]. The AI was prompted to identify systemic disparities, propose structured policy interventions, and iteratively refine recommendations based on the synthesized findings. This approach allowed the generation of standardized, reproducible, and scalable policy suggestions, reducing subjective bias and providing a systematic framework for addressing disparities. The investigators critically review the AI-organized outputs, modify them as needed based on clinical context, and ensure alignment with ethical, practical, and scientific standards. This hybrid approach ensured that all final policy recommendations reflected expert human judgment while leveraging AI’s organizational capabilities to enhance reproducibility and transparency. The AI-assisted component was designed and reported following the TRIPOD- large language models (LLMs) guidelines to ensure transparency, reproducibility, and robustness in leveraging LLMs [26].

1. *Identification of disparities and systemic barriers*
  The AI model was applied to conduct two separate analyses to:
  - Detect disparities in waitlisting, transplantation access, and post-transplant care.
  - Identify policy-driven obstacles affecting insurance-related disparities in the U.S. kidney transplant system.
2. *AI-driven policy recommendations*
  Findings from the AI-assisted analysis informed evidence-based policy recommendations, including:
  - Standardized referral protocols to improve waitlisting equity.
  - Expansion of insurance coverage to enhance access to kidney transplantation.
  - Introduction of financial assistance mechanisms to support immunosuppressive medication adherence.
  - Integration of digital health tools to mitigate gaps in post-transplant monitoring.

The AI-assisted analysis provided a scalable framework for addressing policy gaps and healthcare inequities, complementing traditional epidemiological and systematic review methodologies.

#### Verification of AI analysis consistency

To ensure reproducibility and reliability, the AI-assisted analysis was conducted twice, with a one-week interval between assessments. The AI model was prompted with:

‘Act as a stakeholder in kidney transplantation policy and identify key problems and disparities in the system’.

The consistency of AI-generated insights was evaluated by comparing both assessments, confirming stability in the identification of key disparities and policy challenges.

## Results

**Figure 1.**
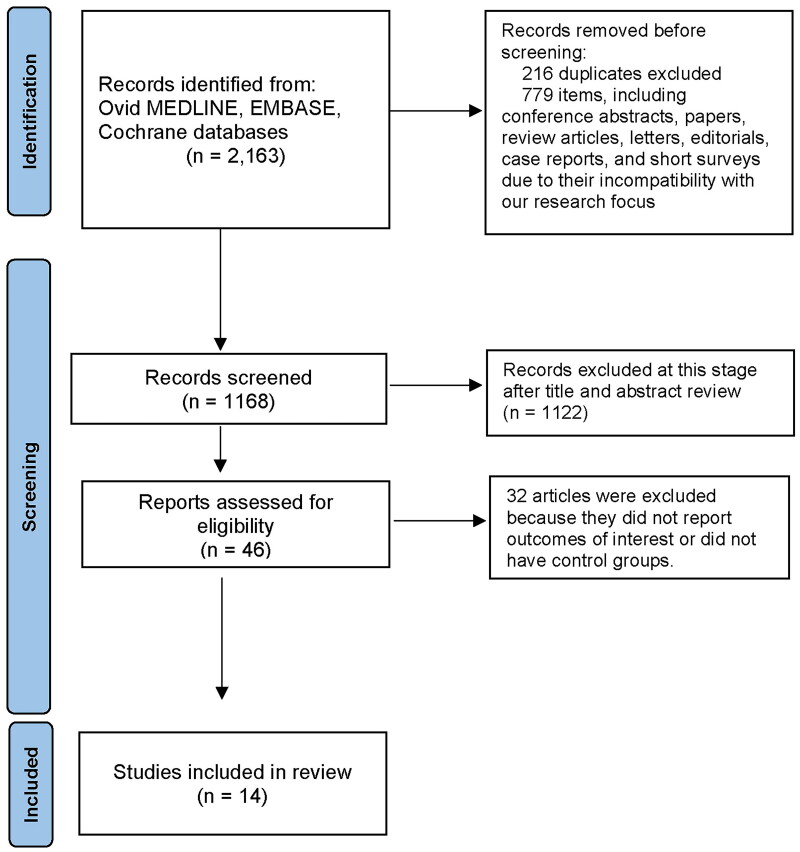
Search methodology and selection process.

Our search strategy initially identified 2163 records. After removing duplicates and irrelevant items, 1168 records were screened, from which 1122 were excluded after title and abstract review due to not meeting our criteria. Further assessment of eligibility led to the exclusion of 32 articles, leaving 14 studies for inclusion in the review (Figure 1 and Tables 1 and 2).

**Table 1.** Baseline characteristic of included studies.

Study	Year published	Database used	Period	Population	Characteristics	Insurance type (%)				NOS score
						Private	Medicare	Medicaid	Other	
Schold et al. [27]	2008	CMS 2728 form	1995–2003	453,429	Adult 18–70 y with initial ESKD Compared listed vs. non-listed within 1-year Sex: male 54.7%, female 45.3% Race: White patients 61%, Black 35%, other 4%	168,004 (36%)	179,567 (39%)	105,858 (23%)	12,282 (2%)	9
Keith et al. [28]	2008	SRTR/OPTS	2001–2004	75,979	Candidate for DDKT Exclude: waitlist LRKT, list for 2^nd^ transplant of any type Two-third of patients aged 40–60 years Sex: male 59%, female 41% Race: White patients 46.3%, Black 29.6%, Hispanic 15.4% Transplant types: Received LRKT 9979 (1.3%) Received DDKT 14,968 (19.7%)	34,115 (44.9%)	33,279 (43.8%)	5774 (7.6%)	5774 (7.6%)	9
Johansen et al. [29]	2012	USRDS, Waitlist Standard Analysis Files	2005–2009	426,489	Adult dialysis patient without prior kidney transplant Categorized into 3 groups: 1. Not informed (unsuitable), 2. Not informed (not assessed), 3. Informed Mean age: 63.2 ± 15.3 years Sex: male 56%, female 44% Race: Black 30%, Hispanic 13.8% Dialysis: HD 93.8%	108,328 (25.4%)	204,432 (47.9%)	47,767 (11.2%)	66,106 (15.5%)	9
Schold et al. [30]	2016	SRTR/OPTN	2007–2014	315,796	Adult patients on waiting list for kidney transplant in 202 transplant centers Compared centers with and without low performance Majority ages 50–64 years Sex: male 60%, female 40% Race: Black 31%, Hispanic 17% Prior kidney transplant 14%	136,132 (43%)	138,191 (44%)	33,088 (10%)	8385 (2.7%)	9
Dubay et al. [12]	2016	SRTR/OPTN	2002–2011	All study 169,194	Adult kidney recipient = 90,548 All population Mean age: 52.2 ± 12.3 years Sex: male 63.4%, female 36.6% Race: White patients 59%, Black 22.1%, Hispanic 12.1% Patient characteristics in kidney recipients: NA Median follow-up time 3.7 (1.9–5.0) years	25,970 (34.7%)	60,217 (75.5%)	4361 (29.9%)	None	9
Harhay et al. 2018 [31]	2018	UNOS	2011–2016	50,598	Adult listed for KT before dialysis dependence Exclude: missing or non-insurance candidates, residents of US territories Compared listing in states with and without Medicaid expansion Mean age: 55 ± 13.5 years Sex: male 57.6%, female 42.4% Race: White patients 59%, Black 18.5%, Hispanic 12% Prior transplant 12.8%	33,873 (66.5%)	13,565 (26.5%)	3520 (7%)	None	9
Hart et al. 2019 [32]	2019	SRTR/OPTN USRDS, Symphony pharmacy fill database	2008–2014	78,861	Adult age < 65 years with Medicare-covered kidney-alone recipient 50% of patients aged 50–65 years Race: White patients 68%, Black 14%, Hispanic 14.2% Transplant type: DDKT 66.5%, LRKT 33.5%	None	78,861 (100%)	None	None	8
King KL et al. [33]	2019	SRTR	2000–2018	157,073	Adulte DDKT recipients after implementation of KAS Compared pre-KAS and post-KAS Mean age 53 ± 13 years Sex: male 60%, female 40% Race: White patients 43%, Black 33%, Hispanic 16% Transplant type: Preemptive DDKT 9.3% Non-preemptive DDKT 90.7%	41,440 (26.4%)	103,350 (65.8%)	8278 (5.3%)	4005 (2.5%)	9
Lenihan et al. [34]	2019	USRDS	1998–2004	101,322	Medicare-insured patients undergoing first kidney transplant with uninterrupted Medicare at least six months and at least one Medicare claim prior transplant Mean age 49 ± 13.8 years Sex: male 62.6%, female 38.4% Race: White patients 60%, Black 32.2%, Other 7.9% Dialysis types: HD 83%, PD 16.3% Prior transplant: 3.1%	None	101,322 (100%)	None	None	9
Ng et al. [9]	2020	UPMC transplant center	2010–2018	1055	Prospective cohort in UPMC KT candidates Compared White patients and African-American KT candidates Mean age 56.7 ± 13.4 years Sex: male 51.6%, female 38.4% Race: White patients 74.7%, Black 25.3%, Transplant type: living donor 52.8%	277 (26.5%)	Public 370 (35.4%)		Both private and public 399 (38.2%)	9
Wesselman et al. [35]	2021	UPMC transplant center	2010–2012	363	Receiving kidney transplant Exclude: previous kidney transplant Mean age: 52 ± 14 years Sex: male 61%, female 39% Race: White patients 83%, Black 18% Dialysis type: HD 55%, PD 10%, none 35%	98 (27%)	Public 148 (41%)		Both private and public 113 (31%)	9
Balakrishnan et al. [36]	2022	Transplant Center in Chicago	2018–2019	234	Cross-sectional study, receiving kidney transplant Mean age: 50.6 ± 11.8 years Sex: male 58%, female 42% Race: White patients 51.3%, Black 25.2%	69 (29.5%)	Public 62 (26.5%)		Both private and public 103 (44%)	9
Morenz et al. [14]	2023	SRTR	2008–2021	247,335	Adult > 17 years, US citizen/resident, on waitlist for transplant Exclusion: retransplant, waitlist for multiorgan transplant Mean age: 55 ± 9.5 years Sex: male 62%, female 38% Race: White patients 40%, Black 31%, Hispanic 19%, Asian 7.8% Year on dialysis: < 1 yr.=5.2%, 1–5 yr.=42%, >5 yr.=30%, non-dialysis 23%	105,360 (42.6%)	Public 141,975 (57.4%)		None	9
Shawwa et al. [37]	2024	USRDS	1965–2020	23,055	Incident ESKD patient in West Virginia Mean age 62.6 ± 15.4 year Sex: male 44.4%, female 45.6% Race: White patients 91%, Black 8.5%, Other 0.7% Dialysis types: in-center HD 83.3%, PD 13.2%, preemptive KT 2.3%, Home HD 0.7% Receiving kidney transplant (1784) 13%	4549 (20%)	12,546 (56%)	4695 (21%)	3448 (16%)	9

Abbreviations: CMS: Centers for Medicare and Medicaid Services; KAS: Kidney Allocation System; MPR: Medication procession ratio; NA: not available; DDKT: deceased related kidney transplant; LRKT: living related kidney transplant; HD: hemodialysis; PD: peritoneal dialysis; KT: kidney transplantation; sd: subdistribution.

**Table 2.** Outcomes of included studies.

Study	Waiting list related	Kidney transplantation	Mortality/survival	Graft failure	Immunosuppressant adherence
Schold et al. [27]	Transplant listing: Listed 11% (49,422): private 62%, Medicare 20%, Medicaid 18% Not listed 89% (404,007): private 34%, Medicare 42%, Medicaid 24% Not listing for transplant within 1 year after ESKD onset (Ref. Medicare), Adjusted OR (95% CI) Private: 0.57 (0.55, 0.59) Medicaid: 1.01 (0.97, 1.05)	Listed and transplant with >5 years life expectancy*: LRKT at 3-year following ESKD: private 27%, public 9% DDKT at 3-year following ESKD: private 33%, public 15% *Age range 18–49 and 59–60 years	Survival on dialysis following ESKD onset (Ref. Medicaid), (95% CI) Private 0.13 (0.11, 0.14), *p* < .001 Medicare −0.05 (−0.07, −0.04), *p* < .001 Other 0.16 (0.15, 0.17, *p* < .001) *Censored transplantation, death, last follow-up time	NA	NA
Keith et al. [28]	Duration of dialysis before listing (Ref. private), days (95% CI): Medicare: 462 (447, 477), *p* < .001 Medicaid: 219 (192, 245), *p*, .001 Listed KT within one year: Private 32%, Medicare 8%, Medicaid 14%, other 27% Preemptive listing, (Ref. private), Adjusted OR (95% CI); Medicare 0.22 (0.21, 0.23), *p* < .001 Medicaid 0.53 (0.49, 0.58), *p* < .001	NA	NA	NA	NA
Johansen et al. [29]	Being reported as not assessed for transplant (Ref. Private), adjusted OR (95% CI) Medicare 1.21 (1.18, 1.24) Medicaid 1.33 (1.28, 1.38) None 1.38 (1.33, 1.44) Transplant waiting list in first year, Adjusted HR (95% CI) Medicare 0.63 (0.61, 0.65) Medicaid 0.52 (0.50, 0.54) None 0.42 (0.41, 0.44)	Transplantation in first year, Adjusted OR (95% CI) Medicare 0.53 (0.49, 0.58) Medicaid 0.40 (0.36, 0.44) None 0.30 (0.26, 0.34)	NA	NA	NA
Schold et al. [30]	Rate of waitlist removal per 1000 follow-up years, (95% CI) Private 36.8 (36.1, 37.5) Medicare 56.4 (55.1,57.3) Medicaid 66.8 (65.0, 68.6) Waitlist removal* and low-performance evaluation, Adjusted HR (95% Ci) Medicare 1.17 (1.14, 1.20) Medicaid 1.61 (1.55, 1.67) Other 1.29 (1.20, 1.38) Low-performance evaluation with waiting list removal was higher in patients with Medicaid as a primary insurance at the time of listing AHR 1.92 (95% CI 1.82–2.04, *p* < .001) *reason for ‘too sick’ or ‘other’	NA	NA	NA	NA
Dubay et al. [12]	Dialysis time before KT, years (95% CI) Total 3.2 (1.5, 5.2), *p* < .001 Private 1.8 (0.5, 3.1), *p* < .001 Medicare 3.8 y (2.2, 5.7), *p* < .001 Medicaid 3.9 y (1.9, 6.4), *p* < .001	Receiving transplantation: private 28.7%, Medicare 66.5%, Medicaid 4.8%	5-year survival after KT (*p* < .001) Private 84.6%, Medicare 78%, Medicaid 82.9% Mortality after KT (Ref. Private), Adjusted HR (95% CI) Medicare 1.36 (1.30, 1.42), *p* < .001 Medicaid 1.37 (1.24, 1.51), *p* < .001 *Adjusted age, sex, race, year on dialysis, Diabetes Mellitus, Hypertension, Creatinine, transplant years	NA	NA
Harhay et al. [31]	Preemptive Medicaid–covered listing in the post-expansion period increased in Expanded Medicare state (59% vs 8.8%) Preemptive listing compared expansion with non-expansion states, proportional difference % (95% CI) Private −4.7 (−6.1 to −3.1), *p* < .001 Medicare 1.4 (0.04–2.8), *p* < .05 Medicaid 3.2 (2.7–3.8), *p* < .001 Proportion of preemptive listing with Medicaid by Race/Ethnicity in Expansion states: White patients 1.4% (4.3% − 5.7%), *p* < .001 Black 4% (11.1% − 15.1%), *p* < .001 Hispanic 5.9% (14.5% − 20.4%), *p* < .001	NA	NA	NA	NA
Hart et al. [32]	Timing of Medicare loss: Early (before 3 years) 2.4% On time (withing 3 years) 39.2% Late (more than 3 years) 7.7%	NA	NA	Graft failure: Early Medicare loss before 3 years was associated with a 10.9 to 17.3 times Late Medicare a loss more than 3 years was associated with a higher hazard of graft failure 2.4–8.4 times	Posttransplant 68% (53,611) had at least one fill for immunosuppressant medication in database Subsequent MPR: Early Medicare loss associated with lower subsequent MPR for all immunosuppressive types compared with non-Medicare loss
King et al. [33]	NA	Preemptive transplantation (Ref. Private), Adjusted OR (95% CI) Pre-KAS: Medicare 0.26 (0.25–0.27), *p* < .001 Medicaid 0.71 (0.64–0.78), *p* = .12 Other 0.81 (0.71–0.93), *p* = .02 Post KAS Medicare 0.20 (0.18–0.22), *p* < .001 Medicaid 0.67 (0.57–0.78), *p* < .12 Other 0.62 (0.53–0.73), *p* = .02 *Adjusted demographic, education factors, transplant-related factors	NA	NA	NA
Lenihan et al. [34]	NA	Hospitalization for: Cardiovascular: 6896 (2.3%), sdHR 0.51 (0.43–0.6) Cancer: 2865 (2.8%), sdHR 1.46 (1.1–1.94) *Adjusted demographic and dialysis-related factors	Mortality 29.6% (30,023) Median follow-up time from transplant to death: 5.5 (2.5–9.1) year adjusted HR 0.46 (95% CI 0.39–0.55)	Graft failure with nonfunctioning graft: 23.3% (23,633), sdHR 0.45 (95% CI 0.3–0.6) Median time of graft failure 4.5 (1.9–7.9) year	NA
Ng et al. [9]	Candidates race/ethnicity: White patients: private 28.5%, public 31.4%, both 40% Black: private 20.5%, public 47.2%, both 32.3% Years to wait-listing for KT (Ref. private), sdHR (95% CI) Public 0.63 (0.49, 0.80), *p* = .002 Both public/private 0.79 (0.63, 0.97), *p* = .028 *Adjusted ethnicity, demographic, medical factors, cultural, psychosocial, and transplant knowledge	NA	NA	NA	NA
Wesselman et al. [35]	NA	Receiving any transplant (ref. private), sd HR (95% CI) Public 0.60 (0.44–0.80) Both public/private 0.67 (0.52–0.88) Receiving LRKT (Ref. private), sdHR (95% CI) Public 0.32 (0.17–0.60) Both Public/Private 0.46 (0.28–0.75) * Adjusted: race/ethnicity, demographic, medical health factor, after KAS, cultural, psychosocial, transplant knowledge, education factor	NA	NA	NA
Balakrishnan et al. [36]	NA	NA	NA	NA	8% of dual public and private insurance had non-adherence risk Immunosuppressant non-adherence risk (Ref. Private), OD (95% CI) Private/Public 5.50 (1.43, 17.82), *p* < .05 Public 2.27 (0.5, 10.31), *p* > .05
Morenz et al. [14]	Removal from the wait list the reason died or become too sick (Ref Private) Public: sdHR 1.33 (1.30–1.36)	Total receiving KT: 42.6% (105,346) Public 69%, private 31% Received DDKT: Public 33% vs Private 26% with sdHR 1.57 (1.54, 1.60) Receive LRKT: 7.8% vs 20% with sdHR 0.87 (0.85, 0.89)	Post-KT mortality rate: Public 9.2%, private 4.0% with RR 1.22 (95% CI 1.15, 1.31)	Allograft failure: Public insurance 5.8%, private 3.7% with RR 11.10 (95% CI 1.03, 1.29)	NA
Shawwa et al. [37]	Waitlist in country-level poverty rate, OR (95% CI) Private 1.66 (1.39, 1.98), *p* < .001 Medicaid 0.54 (0.44, 0.67), *p* < .001 Medicare 0.75 (0.62, 0.9), *p* < .001	Being transplanted, OR (95% CI) Private 2.3 (1.88, 2.8), *p* < .001 Medicaid 0.46 (0.36, 0.58), *p* < .001	NA	NA	NA

Abbreviations: KAS: Kidney Allocation System; KT: kidney transplant; MPR: medication procession ratio; NA: not available; DDKT: deceased related kidney transplant; LRKT: living related kidney transplant; HD: hemodialysis; PD: peritoneal dialysis; KT: kidney transplantation; sd: subdistribution; HR: hazard ratio; OR: odd ratio.

**Figure 2.**
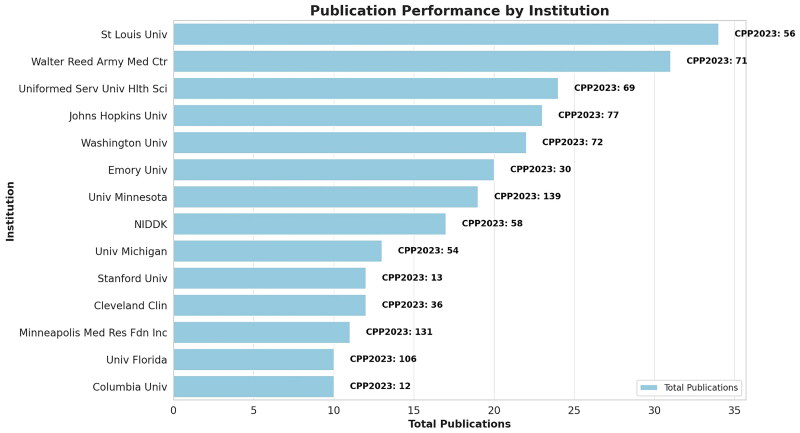
Publication performance by institution. Bar plot representing total publications, while annotations for citations per publication in 2023 (CPP2023) are clearly marked next to each institution in black. This adjustment ensures that the key information—both the volume of publications and the impact of these publications as indicated by CPP2023—is immediately accessible and easy to interpret, highlighting the significant contributions and research impact of the top 14 institutions in the USA within the medical research field.

- Research in Surgery and Transplantation fields is most prolific, with these categories showing the highest number of documents and significant citation impacts, indicating their leading roles in medical research (Supplementary Table 1).
- Key journals identified, such as the American Journal of Transplantation and the Clinical Journal of the American Society of Nephrology, have high impact factors and citations, highlighting their importance in disseminating impactful research (Supplementary Table 2).
- The United States dominates the research output with the majority of publications and citations, emphasizing its central role in the global research environment. Canada also shows notable contributions, especially in terms of international collaboration (Supplementary Table 3).
- Among the top 14 U.S. institutions, St. Louis University and Walter Reed Army Medical Center are leading in terms of publication numbers and citation impact, underlining their significant contributions to medical research (Figure 2, Supplementary Table 4).

**1. Disparities in waitlisting and access to kidney transplantation**

Several studies, including those by Keith et al. [28], Wesselman et al. [35], Harhay et al. [31], and Ng et al. [9] consistently demonstrate disparities in waitlisting for kidney transplantation based on factors including race, insurance status, income level, and education level. These disparities are especially pronounced among African American patients and those with public insurance, such as Medicaid. Insurance status played a pivotal role, with several studies showing worse outcomes for publicly insured patients dependent on Medicare or Medicaid. Publicly insured dialysis patients had a 33% higher likelihood of not being assessed for transplantation compared to privately insured patients in one study [29]. Beyond race and insurance status, lower income and education levels were also associated with reduced probabilities of preemptive listing, increased time to listing, and lower likelihood of transplantation [38,39]. One study highlighted the factors, including income, insurance type, comorbidities, previous transplant history, social support, transplant knowledge, and others that collectively influenced eligibility and access [38].

**2. Post-transplant outcomes and insurance type**

Dubay et al. [12], Lenihan et al. [34], and Morenz et al. [14] reveal significant correlations between insurance type and post-transplant outcomes. Patients with public insurance, such as Medicare and Medicaid, face higher risks of post-transplant mortality and allograft failure. The study by Hart et al. [32] further emphasizes the impact of Medicare loss on medication adherence and subsequent allograft loss.

The studies demonstrated notable disparities in post-transplant outcomes, especially patient and allograft survival rates, based on insurance status. Publicly insured transplant recipients dependent on Medicare or Medicaid consistently had worse outcomes compared to those with private insurance. For instance, Medicare insurance was associated with a 1.4 to 4.9% lower five-year patient survival rate for kidney transplants compared to private insurance (96.9% survival for private insurance vs 92.0% for Medicare), with 37 to 76% higher likelihood of mortality.

The studies emphasized that the loss or transition of insurance coverage post-transplantation seems to exacerbate clinical risks. Early loss of Medicare coverage for transplant recipients under age 65 was linked to 11 to 17 times higher likelihood of graft failure and over 50% reduced immunosuppressive medication adherence. Loss of insurance jeopardizes affordability and continuity of care. While observational and retrospective in nature, the analyses underscored continuing post-transplant insurance-related outcome disparities and the need for targeted policy and healthcare interventions.

**3. Socioeconomic factors and their influence on transplant outcomes**

Studies by Keith et al. [28], Laurentine et al., and Wesselman et al. [35] highlight the influence of socioeconomic factors, including education and income level, on transplant outcomes. These factors are intricately linked with insurance type and contribute to disparities in preemptive transplant listings and access to transplantation. The research strongly indicated that socioeconomic factors like income, education level, and social support have a notable impact on access to transplantation and post-transplant outcomes. Lower income was consistently associated with a reduced likelihood of transplant waitlisting and preemptive listing prior to requiring dialysis. One study highlighted a direct correlation between rising income quintiles and greater probability of transplantation. Education level also mattered - college-educated minority candidates had double the likelihood of preemptive listing versus high school or lesser-educated candidates.

Beyond access issues, socioeconomic disadvantages were linked to worse post-transplant results. An analysis across over 13,000 kidney recipients found that lower neighborhood socioeconomic status was significantly associated with a higher risk of graft failure and mortality independent of race or ethnicity. Recipients in neighborhoods with high school or lesser aggregate education had around 30% higher likelihood of graft loss. This aligns with other findings indicating reduced survival rates for publicly insured recipients who often face socioeconomic constraints.

**4. Impact of healthcare policy and center performance**

Harhay et al. [31] and Schold et al. [30] discuss the influence of healthcare policy, such as Medicaid expansion under the ACA, and transplant center performance on transplantation outcomes. Low-performing centers exhibit higher rates of waitlist removal, underscoring the need for quality improvement in transplant care delivery. The research indicated that changes in national allocation policies and variability in transplant center performance contribute to disparities in access and outcomes. After the 2014 Kidney Allocation System (KAS) changes prioritizing pediatric candidates, disparities widened between publicly and privately insured recipients in terms of preemptive transplantation rates. Centers labeled as ‘low performing’ based on metrics like waitlist removal and mortality rates were associated with faster removal of candidates from the kidney transplant waitlist.

These effects interacted with patient demographics, with Hispanic candidates and those without private insurance seeing stronger associations between low-performing centers and removal from the waitlist without transplantation. However, removed candidates at low-performing centers also had lower post-removal mortality rates, indicating potential center-specific practices around patient selection. Patients with public insurance and ethnic minorities seem especially impacted by both national policy changes and center-specific procedural variations.

Overall, the studies emphasize that healthcare policies and inconsistent center practices can propagate disparities in access and outcomes. While delays may protect some patients, accelerated waitlist removal at low-performing centers has implications for survival probabilities among vulnerable groups. Standardization of center practices and tailored policy adjustments are important to address disproportionate impacts. Improving early education and post-transplant medication coverage can also help address socioeconomic disparity amplification stemming from policy shifts.

**5. Medication adherence and insurance complexity**

The study by Balakrishnan et al. [36] brings attention to the challenge of medication adherence, particularly among patients with dual private/public insurance. This complexity in insurance coverage is linked to poorer immunosuppressant adherence, highlighting the need for simplified and more accessible healthcare models. The research highlighted that insurance coverage complexity, stemming from scenarios like transitioning from one insurance type to another, poses barriers to optimal post-transplant medication adherence. One study [36] found that kidney transplant recipients with dual private and public insurance had a 5.5 times higher likelihood of immunosuppressant non-adherence risk compared to only private coverage. This aligns with other findings showing significantly reduced medication possession ratios for recipients who lost Medicare coverage early after transplantation [32].

Insurance intricacy adds confusion around coverage, formulary restrictions, and out-of-pocket costs for essential post-transplant regimens. Medicare coverage loss for recipients under 65 was linked to over 50% lower medication adherence across immunosuppressant drug classes. The higher non-adherence risk then translates into worse clinical outcomes like higher rates of organ rejection. Beyond insurance complexity, lower health literacy, poorer health status, and higher depression scores were also associated with medication non-adherence.

### Quality assessment

All the eligible cohort studies [9,12,14,27–35,37] had NOS scores of 9, which determined good quality. One cross-sectional study [36] has scored 9 was defined as very good studies. The NOS score of individual studies is presented in Supplementary Table 5.

Key Problems and Disparities in U.S. Kidney Transplantation Policy Analyzed and Identified by o3-mini-high (2025): The AI-assisted analysis validated known disparities in access and outcomes and contributed by structuring the policy recommendations into a comprehensive framework organized by disparity domains (waitlisting, outcomes, socioeconomic barriers, center variability, and medication adherence). Although many of the AI-suggested interventions aligned with previously proposed policies, the model ensured systematic, reproducible prioritization without manual narrative bias, enhancing the scalability of translating evidence into actionable strategies.

**1. Disparities in waitlisting and access to kidney transplantation**

**Key issues:**

- **Racial and ethnic disparities:** African Americans, Hispanics, and other minority groups are significantly less likely to be placed on the transplant waitlist than White patients. Studies by Keith et al. [16], Wesselman et al. [23], and Harhay et al. [19] demonstrate that Black patients have lower rates of referral, evaluation, and waitlisting for kidney transplants.
- **Insurance-based inequities:** Patients with Medicaid or Medicare have a lower likelihood of being evaluated for transplantation compared to those with private insurance. One study found that Medicaid patients were 33% less likely to be assessed for transplantation than privately insured patients.
- **Socioeconomic barriers:** Lower income and education levels are associated with reduced access to preemptive transplantation, longer wait times, and lower transplantation rates. The lack of transplant knowledge and support structures among lower-income groups further exacerbates this issue.

**Proposed policy solutions:**

- **Standardized guidelines** for transplant referrals to ensure equitable access across racial and socioeconomic groups.
- **Expansion of Medicaid coverage** for pre-transplant evaluations and referrals.
- **Increased outreach and education programs** targeting minority and low-income communities.
- **Data-driven monitoring:** Establish a national dashboard to track waitlisting rates, referral patterns, and evaluation outcomes stratified by race, ethnicity, and socioeconomic status to facilitate accountability and rapid policy adjustments.
- **Incentivizing equity:** Introduce performance-based incentives for transplant centers that demonstrate significant improvements in equitable referral and waitlisting practices.
- **Community health workers:** Integrate culturally and linguistically aligned patient navigators to bridge the knowledge gap and assist underserved patients through the referral process.

**2. Post-transplant outcomes and insurance-related disparities**

**Key issues:**

- **Insurance-related survival differences:** Research by Dubay et al. [11], Lenihan et al. [22], and Morenz et al. [13] shows that kidney transplant recipients with Medicare or Medicaid face higher risks of post-transplant mortality and allograft failure compared to privately insured patients.
- **Impact of Medicare loss:** The study by Hart et al. [20] emphasizes that early Medicare loss is associated with lower immunosuppressant adherence and significantly higher graft failure rates.
- **Five-year survival rates:** Medicare patients had a 1.4%–4.9% lower five-year survival rate compared to those with private insurance (96.9% vs. 92.0%).

**Proposed policy solutions:**

- **Expand Medicare coverage** to include broader post-transplant medical services beyond immunosuppressive medications, addressing gaps in routine outpatient care, hospitalization coverage, and management of transplant-related complications. While the Medicare Part B Immunosuppressive Drug (Part B-ID) benefit now provides lifelong coverage for immunosuppressive medications, additional medical services remain uncovered, creating financial barriers that can adversely affect transplant outcomes.
- **Reduce financial barriers** to transplant medications through federal assistance programs.
- **Implement policy measures** to prevent insurance loss from disrupting post-transplant care.
- **Insurance coordination programs:** Develop integrated care models that actively manage insurance transitions, including case management systems that coordinate between private insurers, Medicare, and Medicaid.
- **Outcome-based reimbursement:** Consider reimbursement models that reward transplant centers based on improved post-transplant outcomes to incentivize quality care across insurance types.
- **Pilot programs:** Launch pilot initiatives in high-disparity regions to test comprehensive insurance navigation services that combine financial counseling with medical oversight.

**3. Socioeconomic factors affecting transplant outcomes**

**Key issues:**

- **Lower income linked to worse outcomes:** Studies by Keith et al. [16] and Wesselman et al. [23] highlight that patients from lower socioeconomic backgrounds are less likely to receive transplants and more likely to experience graft failure.
- **Education disparities:** College-educated minority candidates are twice as likely to be preemptively listed compared to those with only a high school education, underscoring the role of education in transplantation success.
- **Neighborhood and community influence:** Living in a low-income neighborhood correlates with higher mortality and graft failure rates, even when controlling for race and insurance status.

**Proposed policy solutions:**

- **Expansion of financial support programs** for transplant candidates from low-income backgrounds.
- **Strengthening community-based education initiatives** on transplant eligibility and post-transplant care.
- **Addressing social determinants of health** in transplant policy reform.
- **Targeted funding initiatives:** Create grants or subsidies specifically for low-income patients to cover direct medical costs and ancillary expenses such as transportation and lodging for evaluations.
- **Local partnerships:** Collaborate with community organizations and local governments to develop tailored education programs, including multilingual outreach and culturally appropriate materials.
- **Integrated social services:** Embed social workers within transplant centers to connect patients to broader support services (housing, nutrition, mental health) addressing the multifactorial nature of socioeconomic disparities.

**4. Impact of healthcare policy and transplant center performance**

**Key issues:**

- **Medicaid expansion improved access:** The study by Harhay et al. [19] found that states that expanded Medicaid under the Affordable Care Act (ACA) saw increased preemptive listings for kidney transplants. However, disparities persist in non-expansion states.
- **Transplant center performance disparities:** Research by Schold et al. [18] demonstrates that low-performing transplant centers are more likely to remove patients from waitlists, disproportionately affecting minorities and Medicaid recipients.
- **Center-level variability:** Policies and procedures for waitlist management vary significantly across transplant centers, leading to inconsistent patient outcomes.

**Proposed policy solutions:**

- **Standardizing waitlist management criteria** across transplant centers to minimize disparities.
- **Strengthening Medicaid expansion** to ensure uniform transplant access across all states.
- **Implementing federal oversight** to improve performance at underperforming transplant centers.
- **Uniform quality metrics:** Develop and mandate a standardized set of quality metrics for all transplant centers, including equity measures such as waitlist diversity and retention rates.
- **Public reporting:** Require regular public reporting of transplant center performance data to foster competition and motivate improvements.
- **Best practice sharing:** Establish a national forum where high-performing centers can share strategies with underperforming centers to accelerate the adoption of effective practices.

**5. Medication adherence and insurance complexity**

**Key issues:**

- **Insurance transitions and medication adherence:** The study by Balakrishnan et al. [24] found that patients with dual private and public insurance had a 5.5 times higher risk of immunosuppressant non-adherence compared to those with private insurance alone.
- **Medicare loss and medication affordability:** Early Medicare loss among transplant recipients under 65 years old is associated with over 50% reduced adherence to immunosuppressive medications, increasing the risk of rejection and graft failure.
- **Healthcare navigation challenges:** Many transplant recipients struggle with navigating insurance coverage changes post-transplantation, leading to lapses in medication adherence.

**Proposed policy solutions:**

- **Simplifying insurance transition processes** for transplant patients.
- **Expanding Medicare coverage** to include lifelong immunosuppressant medication support.
- **Enhancing patient assistance programs** for medication affordability.
- **Digital health interventions:** Implement mobile applications and telemedicine follow-ups to remind patients of medication schedules, track adherence, and provide real-time support.
- **Patient navigator programs:** Establish dedicated roles to assist transplant recipients with managing insurance changes, accessing medications, and scheduling appointments.
- **Integrated care pathways:** Develop comprehensive care pathways that ensure seamless transitions between insurance coverage phases with regular check-ins on medication adherence.

## Conclusion and call to action

The U.S. kidney transplantation system faces critical disparities in access, post-transplant survival, and healthcare equity. Disadvantaged populations—particularly racial minorities, low-income individuals, and publicly insured patients—experience significantly lower rates of transplant waitlisting, poorer post-transplant outcomes, and greater financial barriers to care. Addressing these disparities requires policy reforms focused on equitable access, standardized transplant center practices, expanded insurance coverage, and financial assistance for medication adherence (Table 3).

**Table 3.** AI-suggested policy priorities to address disparities in kidney transplantation (o3-mini-high, 2025).

Next steps for stakeholders	Description
Advocate for Medicare expansion	Ensure post-transplant medication coverage is extended beyond current limitations.
Implement standardized transplant evaluation criteria	Establish uniform guidelines across all transplant centers to ensure equitable patient assessment.
Strengthen financial assistance programs	Expand support for lower-income patients to reduce financial barriers to transplantation and post-transplant care.
Increase education and outreach efforts	Enhance awareness in underserved communities about transplantation, eligibility, and long-term care.
Ensure policy consistency across states	Address geographic disparities by harmonizing state-level policies for equitable transplant access.

## Discussion

This systematic review builds upon and updates the existing literature by incorporating studies conducted during and after major healthcare reforms such as Medicaid expansion under the Affordable Care Act and the introduction of Medicare Part B immunosuppressive drug coverage in 2023. Prior systematic reviews largely provided descriptive summaries of disparities without offering structured policy solutions or accounting for recent shifts in insurance coverage and transplant policies. Our review addresses these limitations by synthesizing the latest available evidence, identifying persistent and emerging inequities, and integrating an AI-assisted, investigator-led framework to systematically generate actionable policy recommendations. This approach represents an advancement over earlier reviews by providing a reproducible, scalable model for translating evidence into targeted interventions aimed at improving transplant equity [12,35,40,41].

Publicly insured patients are significantly less likely to be referred for transplant evaluations and to achieve preemptive waitlisting, a finding that aligns with prior studies identifying systemic biases in referral practices [29,42–44]. These inequities extend into the post-transplant phase, where Medicare and Medicaid recipients exhibit higher mortality and allograft failure rates [32,45,46]. One major concern is the loss of Medicare coverage after 36 months, which has been linked to reduced adherence to immunosuppressive regimens and an elevated risk of graft rejection [32,47,48]. Fortunately, as of 1 January 2023, Medicare has extended coverage for immunosuppressive drugs beyond the previous 36-month limit for kidney transplant recipients. This policy change allows for lifetime coverage of these essential medications under the new Medicare Part B Immunosuppressive Drug (Part B-ID) benefit. However, this extended coverage applies exclusively to immunosuppressive drugs and does not include other Medicare services [49,50].

An unexpected finding of this review was the complexity of dual public-private insurance coverage, which was asso­ciated with poorer immunosuppressant adherence. This contradicts prior assumptions that multiple sources of coverage enhance medication accessibility and underscores the need for targeted financial and policy interventions [5,51]. While Medicaid expansion improved preemptive waitlisting in some states, it did not fully eliminate disparities in transplantation rates or post-transplant outcomes, reinforcing earlier findings on the limitations of state-level policy interventions [31,52,53].

Our methodology intentionally preserved traditional systematic review rigor by completing all steps of evidence identification, selection, data extraction, and quality assessment through manual investigator processes, prior to any AI involvement. The role of the AI model was confined to the policy structuring phase, where it was used to standardize the translation of complex evidence patterns into proposed interventions. All AI outputs were critically reviewed, manually validated, revised, and refined by the investigators through structured consensus discussions. The AI did not independently generate clinical findings, nor did it influence the data synthesis or the prioritization of policy outcomes. This approach ensured that human expertise, judgment, and critical review remained central to all stages of the research process, with AI serving solely as a supportive organizational tool to enhance systematic policy development [54,55].

Our findings build upon and extend prior research by systematically consolidating evidence on insurance-related disparities in kidney transplantation outcomes and uniquely applying an AI-assisted policy analysis framework [35,38,40,41]. This dual-method approach enables a deeper understanding of how systemic inequities manifest and offers data-driven, scalable policy recommendations to address them. Importantly, while social determinants of health have been well-described, actionable pathways to mitigate these disparities have been less frequently proposed in a structured, reproducible manner. By incorporating AI-driven policy recommendations and digital health interventions, this study builds upon existing literature advocating for systemic reforms to reduce disparities in kidney transplantation [38,56]. The analytical approach not only reinforces established concerns but also provides a novel framework for understanding how policy and institutional structures perpetuate inequitable outcomes, emphasizing the need for targeted intervention strategies.

Several interrelated factors likely contribute to these disparities. Structural barriers, such as lower provider reimbursement rates associated with public insurance, may deter transplant centers from prioritizing Medicaid patients [57,58]. Socioeconomic challenges, including financial constraints, transportation difficulties, and out-of-pocket costs for pre-transplant evaluations and post-transplant medications, further exacerbate inequities [57,58]. Health literacy and navigation challenges among publicly insured patients may contribute to delays in transplant evaluations and listing, limiting access to life-saving treatment [58,59]. Institutional variability across transplant centers further compounds these disparities, as inconsistent policies and practices influence patient selection, referral rates, and waitlist management [60,61].

AI-assisted analytics in this review not only provided additional insights into existing inequities but also generated structured, scalable policy solutions that are directly aligned with ongoing efforts to promote health equity in transplantation. By operationalizing complex data into targeted interventions—such as standardizing referral practices, expanding insurance coverage for immunosuppressants, and integrating digital health solutions—this study offers a pragmatic framework for stakeholders seeking to translate the recognition of disparities into measurable improvements in patient care and outcomes [20,21,62]. AI-driven methodologies present a scalable framework for healthcare policy reform, with applications extending beyond kidney transplantation to broader disparities in organ allocation and transplant access [21,63–65]. However, the use of AI in policy generation has inherent limitations. The ‘o3-mini-high (2025)’ model, employed in this study, was trained and validated on retrospective datasets, which may introduce biases inherent to historical transplant practices. While AI can augment policy recommendations, expert oversight remains essential to ensure that these models align with clinical realities and ethical considerations. Future advancements should focus on refining AI interpretability and incorporating real-time data streams to enhance predictive accuracy and fairness in policy formulation. The AI-assisted analysis in this review did not aim to independently discover novel disparities or radically divergent policies; rather, its primary value lay in systematically synthesizing and structuring policy responses based on complex evidence patterns. Compared to traditional narrative reviews where policy proposals may vary by author emphasis, the AI-driven framework ensured that each disparity domain (access, outcomes, insurance loss, center variability, socioeconomic barriers) was systematically addressed, prioritized, and matched with feasible interventions [21,63–65]. Although the AI-proposed recommendations overlapped substantially with prior proposals, the AI-assisted approach offered advantages in scalability, consistency, reproducibility, and rapid iteration, particularly important for real-time policy updating as transplant equity efforts evolve. For future work, AI frameworks could be further leveraged to perform continuous gap analyses, scenario modeling, and dynamic prioritization of equity interventions based on evolving healthcare landscapes.

AI has the potential to transform health disparities research by operationalizing complex, multidimensional data into actionable insights. Specifically, AI models can (1): systematically identify patterns and interactions among multiple social determinants (e.g., insurance type, income, race, geography) that traditional analyses may overlook (2); perform real-time gap analyses by continuously monitoring disparities as healthcare policies and demographics evolve (3); simulate the impact of proposed policy interventions under different scenarios, enabling dynamic prioritization of strategies; and (4) standardize evidence synthesis and policy generation, reducing narrative bias and ensuring reproducibility. In the context of transplantation, AI-driven frameworks could help allocate resources more equitably, optimize waitlisting practices, predict at-risk populations for graft loss, and forecast the effects of insurance reforms on transplant outcomes over time. By integrating these capabilities, AI can shift disparities research from descriptive reporting to proactive, iterative solution modeling.

### Stakeholder roles and policy change process

Addressing disparities in kidney transplantation requires coordinated action among multiple stakeholders. Government and regulatory agencies, including the United Network for Organ Sharing (UNOS), the Health Resources and Services Administration (HRSA), the Centers for Medicare & Medicaid Services (CMS), and the U.S. Congress, play central roles in shaping transplant policies [66–68]. UNOS, through the Organ Procurement and Transplantation Network (OPTN), is responsible for policy development, while HRSA and CMS enforce compliance with national health objectives and reimbursement policies [66,67,69].

Professional organizations such as the American Society of Transplantation (AST) and the American Society of Transplant Surgeons (ASTS) contribute to the development of clinical guidelines and advocate for policy changes [70,71]. Advocacy groups, including the National Kidney Foundation (NKF), Donate Life America, and the Transplant Recipients International Organization (TRIO), further support these efforts by raising public awareness and lobbying for legislative reforms [72,73]. The policy change process follows a structured cycle involving issue identification, stakeholder consultation, committee review, and final policy approval by governing bodies such as the OPTN Board of Directors [74,75]. The roles of these stakeholders and their respective policy contributions are outlined in Table 4. Recent adjustments to the Kidney Allocation System and Medicaid expansion initiatives underscore the evolving landscape of transplant governance and highlight the importance of continuous policy evaluation [76,77].

**Table 4.** Stakeholders in kidney transplantation policy.

Category	Entity	Role in policy change
Government & regulatory bodies	UNOS	Oversees the transplant system, develops policies through OPTN, and implements policy changes.
	HRSA	Administers and monitors OPTN and SRTR, ensuring compliance with national health goals.
	CMS	Regulates transplant centers and enforces policies through reimbursement conditions.
	U.S. Congress	Passes laws affecting transplant policies, funding, and regulations.
Professional & scientific organizations	AST	Provides clinical guidelines, advocates for policy improvements, and conducts research.
	ASTS	Focuses on transplant surgery policies and works with UNOS/OPTN on procurement and allocation.
	NKF	Advocates for kidney patients, raises awareness, and lobbies for policy improvements.
	SRTR	Collects transplant data and conducts analyses to guide policy decisions.
Industry & advocacy groups	OPOs	Manage organ recovery, coordinate with transplant centers, and implement policies regionally.
	Patient Advocacy Groups (Donate Life America, TRIO)	Represent patients and donors, advocate for legislative changes, and improve transplant access.
Policy change process	OPTN	Identifies issues, gathers public comments, reviews policies through committees, and finalizes through the OPTN Board of Directors.

Abbreviations: AST: American Society of Transplantation; ASTS: American Society of Transplant Surgeons; CMS: Centers for Medicare & Medicaid Services; HRSA: Health Resources & Services Administration; NKF: National Kidney Foundation; OPOs: Organ Procurement Organizations; OPTN: Organ Procurement and Transplantation Network; SRTR: Scientific Registry of Transplant Recipients; TRIO: Transplant Recipients International Organization; UNOS: United Network for Organ Sharing; U.S. Congress: United States Congress.

### Policy recommendations

To promote equitable access to kidney transplantation, this review proposes several key policy actions. Ensuring continuous post-transplant care and preventing gaps in immunosuppressive therapy remains a priority [47]. Fortunately, as of 1 January 2023, Medicare has extended coverage for immunosuppressive drugs beyond the previous 36-month limit for kidney transplant recipients. This policy change allows for lifetime coverage of these essential medications under the new Medicare Part B Immunosuppressive Drug (Part B-ID) benefit [49,50]. However, this extended coverage applies exclusively to immunosuppressive drugs and does not include other Medicare services, such as hospitalizations, routine medical care, or additional post-transplant treatments. While this expansion is a significant advancement, further policy efforts should focus on ensuring comprehensive post-transplant care, addressing financial barriers beyond medication coverage, and improving patient education on available benefits to optimize long-term transplant outcomes [49,50].

Standardizing transplant evaluation criteria across transplant centers would help reduce variability in patient selection and improve consistency in access to care [78]. Expanding financial assistance programs would alleviate the burden of medication adherence challenges among low-income patients, which has been a well-documented contributor to post-transplant disparities [44,79]. Additionally, increasing community-based education programs to improve transplant literacy and awareness, particularly in underserved populations, may enhance early engagement with the transplant system and improve outcomes [80,81]. Harmonizing Medicaid policies across states is also necessary to ensure that geographic disparities do not further limit access to transplantation [82,83]. These proposed policy actions and the responsible stakeholders involved in their implementation are summarized in Table 5.

**Table 5.** Next steps for stakeholders.

Next steps for stakeholders	Description	Responsible stakeholders	How each stakeholder can address this action
Ensure awareness and enrollment in Medicare Part B-ID	Promote awareness and streamline enrollment in the extended Medicare immunosuppressive drug coverage.	CMS, HRSA, NKF, Patient Advocacy Groups (Donate Life America, TRIO)	CMS can enhance outreach efforts for eligible patients. HRSA and NKF can provide educational materials. Advocacy groups can assist in patient enrollment and awareness campaigns.
Expand post-transplant care beyond immunosuppressive coverage	Advocate for broader Medicare coverage, including routine post-transplant care and hospital services.	U.S. Congress, CMS, HRSA, NKF, Patient Advocacy Groups	Congress can pass legislation to include broader post-transplant services under Medicare. CMS and HRSA can develop reimbursement models for long-term transplant care.
Implement standardized transplant evaluation criteria	Establish uniform guidelines across all transplant centers to ensure equitable patient assessment.	UNOS, OPTN, CMS, AST, ASTS	UNOS/OPTN can develop policy mandates for transplant centers. CMS can enforce adherence through reimbursement conditions. AST and ASTS can create evidence-based clinical guidelines.
Strengthen financial assistance programs	Expand support for lower-income patients to reduce financial barriers to transplantation and post-transplant care.	CMS, HRSA, U.S. Congress, NKF, Patient Advocacy Groups	CMS and HRSA can develop new funding models. Congress can allocate financial assistance through legislation. NKF and advocacy groups can provide direct patient support and policy advocacy.
Increase education and outreach efforts	Enhance awareness in underserved communities about transplantation, eligibility, and long-term care.	HRSA, NKF, OPOs, AST, ASTS, Patient Advocacy Groups	HRSA and NKF can fund and implement public education campaigns. OPOs can engage with local communities. AST and ASTS can provide patient-friendly educational resources.
Ensure policy consistency across states	Address geographic disparities by harmonizing state-level policies for equitable transplant access.	CMS, HRSA, UNOS, OPTN, U.S. Congress	CMS and HRSA can ensure Medicaid expansion for transplant services. UNOS/OPTN can standardize transplant policies nationwide. Congress can legislate policies to minimize state-to-state variability.

Abbreviations: AST: American Society of Transplantation; ASTS: American Society of Transplant Surgeons; CMS: Centers for Medicare & Medicaid Services; HRSA: Health Resources & Services Administration; NKF: National Kidney Foundation; OPOs: Organ Procurement Organizations; OPTN: Organ Procurement and Transplantation Network; TRIO: Transplant Recipients International Organization; UNOS: United Network for Organ Sharing; U.S. Congress: United States Congress.

Despite its comprehensive scope, this review has several limitations. Heterogeneity across the included studies represents an important limitation of this systematic review. Sources of heterogeneity included differences in insurance classification (e.g., grouping Medicare and Medicaid vs. separate analysis), demographic composition of study populations, clinical settings (single-center vs. national registry data), study periods spanning significant policy changes (e.g., pre- and post-Affordable Care Act), and varying definitions of outcomes such as graft failure and immunosuppressant adherence. These variations precluded meaningful statistical pooling through meta-analysis. However, the thematic consistency of findings across studies—despite these methodological differences—supports the robustness of the overall conclusions. Future work should aim to standardize outcome definitions and analytical frameworks to reduce heterogeneity and facilitate meta-analytical synthesis in this important area. Most of the studies analyzed relied on retrospective observational designs, limiting causal inferences and introducing potential biases. Additionally, while this review focuses on disparities in the United States, kidney transplantation inequities in the global context remain underexplored and warrant further research [84]. Future studies should prioritize prospective cohort designs to establish causal links between insurance coverage and transplant outcomes. Comparative analyses assessing Medicaid expansion across different states, as well as longitudinal studies evaluating the impact of recent policy changes, would provide valuable insights. Furthermore, intervention-based research on patient navigation programs, community health worker models, and AI-driven decision-support tools could help identify actionable strategies to reduce disparities in transplant access and outcomes.

## Conclusion

Profound disparities persist within the U.S. kidney transplantation system, disproportionately affecting publicly insured patients and individuals from marginalized racial, ethnic, and socioeconomic backgrounds. These disparities manifest as lower rates of transplant referrals, reduced access to preemptive waitlisting, and poorer post-transplant outcomes. Addressing these inequities requires comprehensive policy reforms, the standardization of clinical practices, and enhanced financial and educational support for vulnerable populations. This study applies AI-driven methodologies to establish a robust framework for advancing policy interventions in kidney transplantation. Standardized referral protocols, broader insurance coverage, and increased financial support for post-transplant medication adherence are essential to enhancing equity and clinical outcomes. Coordinated efforts among government agencies, professional societies, and patient advocacy groups present a practical path forward to reform the transplant system and address disparities in access, survival, and healthcare equity.

## Supplementary Material

Online Supplementary 05_2025.docx
