# Supplementary material for: Health insurance and kidney transplantation outcomes in the United States: a systematic review and AI-driven analysis of disparities in access and survival
Source: Ren Fail. 2025 Jun 9;47(1):2513007. doi: 10.1080/0886022X.2025.2513007 (PMC12152983; doi:10.1080/0886022X.2025.2513007)
Supplement: Online Supplementary 05_2025.docx [file IRNF_A_2513007_SM9815.docx]

**Online Supplementary Table 1.** The top 11 most productive Web of Science categories.

| Web of Science Category | *TP* (%) | *APP* | *CPP*_2023_ | No *J* |
| --- | --- | --- | --- | --- |
| Surgery | 120 (47) | 6.3 | 54 | 212 |
| Transplantation | 116 (45) | 6.1 | 55 | 26 |
| Urology and nephrology | 89 (34) | 5.7 | 47 | 88 |
| Immunology | 48 (19) | 6.7 | 37 | 161 |
| General and internal medicine | 20 (7.8) | 6.0 | 90 | 168 |
| Cardiac and cardiovascular systems | 5 (1.9) | 7.8 | 20 | 142 |
| Health care sciences and services | 5 (1.9) | 4.4 | 7.2 | 105 |
| Public, environmental and occupational health | 5 (1.9) | 5.0 | 15 | 207 |
| Pediatrics | 4 (1.6) | 4.8 | 15 | 130 |
| Peripheral vascular disease | 4 (1.6) | 5.3 | 20 | 67 |
| Rheumatology | 4 (1.6) | 9.3 | 14 | 35 |

TP (%): total number of documents as a percentage of the total; No. J: number of journals in a category in 2022; APP: average number of authors per publication; *CPP*_2022_: average number of citations per publication (*TC*_2023_/*TP*); *TC*_2023_: total number of citations from publish year until the end of 2023 from the Web of Science Core Collection.

**Online Supplementary Table 2.** The top 10 most productive journals.

| Journal | *IF*_2022_ | *TP* (%) | *APP* | *CPP*_2023_ | Web of Science Category |
| --- | --- | --- | --- | --- | --- |
| American Journal of Transplantation | 8.8 | 45 (18) | 5.7 | 92 | surgery  transplantation |
| Transplantation | 6.2 | 41 (16) | 6.7 | 40 | immunology  surgery  transplantation |
| Clinical Journal of the American Society of Nephrology | 9.8 | 20 (7.8) | 7.2 | 45 | urology and nephrology |
| American Journal of Kidney Diseases | 13.2 | 15 (5.8) | 6.8 | 76 | urology and nephrology |
| Journal of the American Society of Nephrology | 13.6 | 15 (5.8) | 5.5 | 70 | urology and nephrology |
| Clinical Transplantation | 2.1 | 12 (4.7) | 5.7 | 20 | surgery  transplantation |
| Kidney International | 20 | 7 (2.7) | 4.7 | 74 | urology and nephrology |
| Seminars in Dialysis | 1.6 | 6 (2.3) | 1.7 | 13 | urology and nephrology |
| Transplantation Proceedings | 0.90 | 6 (2.3) | 6.5 | 21 | immunology  surgery  transplantation |
| American Journal of Nephrology | 8.8 | 5 (1.9) | 7.8 | 34 | urology and nephrology |

*TP*: number of all documents; %: percentage of documents; *IF*_2022_: journal’s impact factor in 2022; *APP*: average number of authors per publication; *CPP*_2022_: average number of citations per publication (*TC*_2023_/*TP*)

**Online Supplementary Table 3**. Comparison of publication performance of countries.

| Country | TP | TP (n = 256) | | IP_C_ (n = 233) | | CP_C_ (n = 23) | | FP (n = 256) | | RP (n = 256) | | SP (n = 13) | |
| --- | --- | --- | --- | --- | --- | --- | --- | --- | --- | --- | --- | --- | --- |
|  |  | R (%) | CPP_2023_ | R (%) | CPP_2023_ | R (%) | *CPP*_2023_ | R (%) | CPP_2023_ | R (%) | CPP_2023_ | R (%) | CPP_2023_ |
| USA | 250 | 1 (98) | 51 | 1 (97) | 53 | 1 (100) | 34 | 1 (95) | 51 | 1 (95) | 51 | 1 (92) | 25 |
| Canada | 18 | 2 (7.0) | 27 | 2 (1.7) | 12 | 2 (61) | 31 | 2 (3.9) | 31 | 2 (4.3) | 36 | N/A | N/A |
| Italy | 2 | 3 (0.78) | 36 | N/A | N/A | 3 (8.7) | 36 | N/A | N/A | N/A | N/A | N/A | N/A |
| Poland | 2 | 3 (0.78) | 41 | N/A | N/A | 3 (8.7) | 41 | N/A | N/A | N/A | N/A | N/A | N/A |
| Pakistan | 1 | 5 (0.39) | 5.0 | 3 (0.43) | 5.0 | N/A | N/A | 3 (0.39) | 5.0 | 3 (0.39) | 5.0 | N/A | N/A |
| France | 1 | 5 (0.39) | 103 | N/A | N/A | 5 (4.3) | 103 | 3 (0.39) | 103 | N/A | N/A | N/A | N/A |
| Denmark | 1 | 5 (0.39) | 2.0 | N/A | N/A | 5 (4.3) | 2.0 | N/A | N/A | N/A | N/A | N/A | N/A |
| Australia | 1 | 5 (0.39) | 27 | N/A | N/A | 5 (4.3) | 27 | N/A | N/A | N/A | N/A | N/A | N/A |
| Switzerland | 1 | 5 (0.39) | 47 | N/A | N/A | 5 (4.3) | 47 | N/A | N/A | N/A | N/A | N/A | N/A |
| Germany | 1 | 5 (0.39) | 1.0 | 3 (0.43) | 1.0 | N/A | N/A | 3 (0.39) | 1.0 | 3 (0.39) | 1.0 | 2 (7.7) | 1.0 |
| Israel | 1 | 5 (0.39) | 11 | N/A | N/A | 5 (4.3) | 11 | N/A | N/A | N/A | N/A | N/A | N/A |

TP: total number of documents; TP R (%): the percentage of total documents published by each country; IP_C_ R (%): the rank and percentage of single-country documents in all single-country documents; CP_C_ R (%): the rank and percentage of internationally collaborative documents in all internationally collaborative documents; FP R (%): the rank and percentage of first-author documents in all first-authors documents; RP R (%): the rank and percentage of corresponding-author documents in all corresponding-author documents; SP R (%): the rank and percentage of single-author documents in all single-author documents; CPP_2023_: average number of citations per document (TC_2023_/TP); N/A: data is not available.

**Online Supplementary Table 4**. Comparison of publication performance of the top 14 institutions (all in the USA).

| Institution | TP | TP (n = 256) | | IP_I_ (n = 50) | | CP_I_ (n = 206) | | FP (n = 256) | | RP (n = 253) | |
| --- | --- | --- | --- | --- | --- | --- | --- | --- | --- | --- | --- |
|  |  | R (%) | CPP_2023_ | R (%) | CPP_2023_ | R (%) | *CPP*_2023_ | R (%) | CPP_2023_ | R (%) | CPP_2023_ |
| St Louis Univ | 34 | 1 (13) | 55 | 4 (4.0) | 37 | 1 (16) | 56 | 2 (7.8) | 69 | 1 (9.1) | 64 |
| Walter Reed Army Med Ctr | 31 | 2 (12) | 71 | N/A | N/A | 2 (15) | 71 | 1 (8.6) | 76 | 2 (8.3) | 78 |
| Uniformed Serv Univ Hlth Sci | 24 | 3 (9.4) | 69 | N/A | N/A | 3 (12) | 69 | N/A | N/A | 36 (0.4) | 33 |
| Johns Hopkins Univ | 23 | 4 (9.0) | 77 | N/A | N/A | 4 (11) | 77 | 3 (5.5) | 83 | 5 (3.6) | 38 |
| Washington Univ | 22 | 5 (8.6) | 69 | 1 (6.0) | 46 | 5 (9.2) | 72 | 7 (2.3) | 47 | 8 (2.0) | 53 |
| Emory Univ | 20 | 6 (7.8) | 28 | 1 (6.0) | 19 | 7 (8.3) | 30 | 4 (5.1) | 29 | 3 (5.1) | 28 |
| Univ Minnesota | 19 | 7 (7.4) | 132 | 9 (2.0) | 21 | 6 (8.7) | 139 | 11 (1.6) | 59 | 16 (1.2) | 34 |
| NIDDK | 17 | 8 (6.6) | 58 | N/A | N/A | 7 (8.3) | 58 | 35 (0.39) | 27 | 19 (0.79) | 14 |
| Univ Michigan | 13 | 9 (5.1) | 52 | 4 (4.0) | 39 | 10 (5.3) | 54 | 9 (2.0) | 45 | 13 (1.6) | 45 |
| Stanford Univ | 12 | 10 (4.7) | 13 | N/A | N/A | 9 (5.8) | 13 | 6 (2.7) | 7 | 6 (2.8) | 7 |
| Cleveland Clin | 12 | 10 (4.7) | 34 | 4 (4.0) | 23 | 12 (4.9) | 36 | 5 (3.9) | 28 | 4 (4.0) | 28 |
| Minneapolis Med Res Fdn Inc | 11 | 12 (4.3) | 131 | N/A | N/A | 10 (5.3) | 131 | 16 (1.2) | 97 | 13 (1.6) | 106 |
| Univ Florida | 10 | 13 (3.9) | 86 | 1 (6.0) | 40 | 22 (3.4) | 106 | 9 (2.0) | 46 | 8 (2.0) | 46 |
| Columbia Univ | 10 | 13 (3.9) | 17 | 9 (2.0) | 54 | 13 (4.4) | 12 | 19 (0.78) | 31 | 19 (0.79) | 31 |

TP: total number of publications; TP R (%): The percentage of total articles published by each country; IP_I_ R (%): the rank and percentage of single-institute publications in all single-institute articles; CP_I_ R (%): the rank and percentage of inter-institutionally-collaborative articles in all inter-institutionally collaborative articles; FP R (%): the rank and percentage of first-author articles in all first-authors articles; RP R (%): the rank and percentage of corresponding-author articles in all corresponding-author articles; CPP_2023_: average number of citations per publication (TC_2023_/TP); N/A: data is not available.

**Supplemental Table 5.** Summary of Newcastle Ottawa Scale scores (NOS) for included Cohort and Cross-sectional studies.

| **Author (year)** | | **Selection** | **Comparability** | **Outcome** | **Scores** |
| --- | --- | --- | --- | --- | --- |
| **Cohort study** | |  |  |  | **(Total 9)** |
| Schold et al. (2008) | | 4 | 2 | 3 | 9 |
| Keith et al. (2008) | | 4 | 2 | 3 | 9 |
| Johansen et al. (2012) | | 4 | 2 | 3 | 9 |
| Schold et al. (2016) | | 4 | 2 | 3 | 9 |
| Dubay et al. (2016) | | 4 | 2 | 3 | 9 |
| Harhay et al (2018) | | 4 | 1 | 3 | 8 |
| Hart et al. (2019) | | 4 | 2 | 3 | 9 |
| King KL et al. (2019) | | 4 | 2 | 3 | 9 |
| Lenihan et al. (2019) | | 4 | 2 | 3 | 9 |
| Ng et al. (2020) | | 4 | 2 | 3 | 9 |
| Wesselman et al. (2021) | | 4 | 2 | 3 | 9 |
| Morenz et al. (2023) | | 4 | 2 | 3 | 9 |
| Shawwa et al. (2024) | | 4 | 2 | 3 | 9 |
| **Cross – sectional study** | |  |  |  | **(Total 10)** |
| Balakrishnan et al. (2022) | | 4 | 2 | 3 | 9/10 |
|  |  | |  |  |  |
